# Light-based methods for predicting circadian phase in delayed sleep–wake phase disorder

**DOI:** 10.1038/s41598-021-89924-8

**Published:** 2021-05-25

**Authors:** Jade M. Murray, Michelle Magee, Tracey L. Sletten, Christopher Gordon, Nicole Lovato, Krutika Ambani, Delwyn J. Bartlett, David J. Kennaway, Leon C. Lack, Ronald R. Grunstein, Steven W. Lockley, Shantha M. W. Rajaratnam, Andrew J. K. Phillips

**Affiliations:** 1 Turner Institute for Brain and Mental Health, School of Psychological Sciences, Monash University, 18 Innovation Walk, Clayton, VIC 3800 Australia; 2 Cooperative Research Centre for Alertness, Safety and Productivity, Clayton, VIC Australia; 3 NHMRC Centre for Sleep and Circadian Neurobiology, Sydney, NSW Australia; 4 Centre for Neuroscience of Speech, Department of Audiology and Speech Pathology, University of Melbourne, Melbourne, VIC Australia; 5 Woolcock Institute of Medical Research and Sydney Local Health District, Sydney, NSW Australia; 6 University of Sydney Susan Wakil School of Nursing, Camperdown, NSW Australia; 7 Adelaide Institute for Sleep Health, College of Medicine and Public Health, Flinders University, Adelaide, SA Australia; 8 Robinson Research Institute and School of Medicine, University of Adelaide, Adelaide, SA Australia; 9 Division of Sleep and Circadian Disorders, Departments of Medicine and Neurology, Brigham and Women’s Hospital, Boston, MA USA; 10 Division of Sleep Medicine, Harvard Medical School, Boston, MA USA

**Keywords:** Circadian rhythms and sleep, Diagnostic markers

## Abstract

Methods for predicting circadian phase have been developed for healthy individuals. It is unknown whether these methods generalize to clinical populations, such as delayed sleep–wake phase disorder (DSWPD), where circadian timing is associated with functional outcomes. This study evaluated two methods for predicting dim light melatonin onset (DLMO) in 154 DSWPD patients using ~ 7 days of sleep–wake and light data: a dynamic model and a statistical model. The dynamic model has been validated in healthy individuals under both laboratory and field conditions. The statistical model was developed for this dataset and used a multiple linear regression of light exposure during phase delay/advance portions of the phase response curve, as well as sleep timing and demographic variables. Both models performed comparably well in predicting DLMO. The dynamic model predicted DLMO with root mean square error of 68 min, with predictions accurate to within ± 1 h in 58% of participants and ± 2 h in 95%. The statistical model predicted DLMO with root mean square error of 57 min, with predictions accurate to within ± 1 h in 75% of participants and ± 2 h in 96%. We conclude that circadian phase prediction from light data is a viable technique for improving screening, diagnosis, and treatment of DSWPD.

## Introduction

Humans possess an endogenous circadian clock that is responsible for synchronization of many physiological and behavioral processes^1^. Quantitative techniques, such as mathematical models and machine-learning approaches, have been developed to predict the timing of circadian rhythms from non-invasive ambulatory signals^2^. Commonly used ambulatory signals include activity and light^3–6^, skin temperature^7,8^, and heart rate or heart rate variability^9^. Mathematical models, such as the Jewett-Kronauer model^3^, quantify the characteristics of the circadian clock and its response to light, in particular the phase-dependent sensitivity of the clock to light. These models have been developed and tested in healthy individuals under laboratory and field conditions to predict circadian phase markers such as core body temperature minimum (CBTmin)^3,10^ and dim light melatonin onset (DLMO)^4^. It is currently unknown, however, whether these models can accurately predict circadian phase in clinical populations, such as those with circadian rhythm sleep disorders.

One such disorder, delayed sleep–wake phase disorder (DSWPD), is thought to be driven by an underlying delay in the timing of the circadian clock relative to the required sleep–wake schedule^11,12^. Despite this etiology, current diagnostic criteria do not mandate an objective measure of circadian phase. Rather, diagnosis is based on measures of sleep timing^13^ with delayed timing of sleep onset and wake times considered indicative of a delay in the biological clock^12,13^. This is problematic, as sleep and/or wake times can be delayed despite no underlying circadian delay^14^. For example, we have shown previously that 43% of DSWPD patients, using current diagnostic criteria, do not have a circadian phase delay relative to the desired sleep–wake schedule^14^, with others reporting similar findings^15^. This apparent discrepancy can occur because the relationship between the onset of the evening rise in melatonin (a gold-standard circadian phase marker^16^) and sleep is highly variable, with an inter-individual range of up to 5 h between melatonin onset and sleep onset in healthy populations^17–19^. This range can be even greater in sleep disordered populations, with a difference of up to 8 h seen in patients with insomnia^20^.

Currently, circadian phase assessments using salivary DLMO are not widely applied in clinical practice due to the cost, lack of insurance reimbursement, perceived inconvenience of the procedures, and complicated analytical interpretation of results^21^. While simpler biochemical measures are being developed^22–24^, this clinical gap could be addressed by less invasive techniques that attempt to predict circadian timing using ambulatory monitoring of activity and light, given that light is the primary synchronizing agent for the circadian clock^25^.

Here we evaluated the utility of a statistical regression model for predicting circadian phase in a sample of patients clinically diagnosed with DSWPD. To evaluate the performance of the model, we compared its performance against a dynamic model^3^ that has been shown to accurately predict circadian phase in healthy individuals^4,10^.

## Results

### Participant characteristics

Participants (N = 154) were 30.1 ± 10.7 years of age (range 16–64 years) with body mass index (BMI) of 24.6 ± 4.0 kg/m^2^. Average DLMO time was 22:07 (range 18:42–2:24), average bedtime was 0:40, and average wake time was 8:44 (see Table 1). There were no significant differences between the test and training datasets for sex, age, DLMO time, desired bedtime-DLMO phase angle, bed and wake times, composite morningness–eveningness questionnaire (cMEQ), or clinical global impression (CGI) scale. BMI differed modestly between the training and test datasets (23.8 vs. 25.3 kg/m^2^), although both groups were still within the healthy range.

**Table 1:** Participant characteristics of the overall, training and test datasets.

	Overall data set	Training set	Test set	*p*
N	154	77	77	
Sex n (%)	70 M (45.5), 84 F (54.5)	31 M (40.3), 46 F (59.7)	39 M (50.6), 38 F (49.4)	0.20
Age (y) M ± SD	30.1 ± 10.7	29.6 ± 11.4	30.5 ± 10.1	0.61
BMI (kg/m^2^) M ± SD	24.5 ± 4.0	23.8 ± 3.6	25.3 ± 4.2	**0.02**
DLMO time (hh:mm), M ± SD	22:07 ± 1:26	22:11 ± 1:22	22:04 ± 1:31	0.61
DBT-DLMO PAD (h), M ± SD	− 0:19 ± 1:17	− 0:11 ± 1:15	− 0:25 ± 1:18	0.28
Bedtime (hh:mm), M ± SD*	0:41 ± 1:19	0:41 ± 1:13	0:40 ± 1:24	0.94
Wake time (h:mm), M ± SD*	8:46 ± 1:21	8:49 ± 1:17	8:42 ± 1:26	0.71
**CGI**^**˄**^				
Mildly ill	74 (48.7)	31 (40.8)	43 (56.6)	0.05
Moderately-severely ill	78 (51.3)	45 (59.2)	33 (43.4)	
**cMEQ**				
Moderately evening	23 (14.9)	8 (10.4)	15 (19.5)	0.11
Definitely evening	131 (85.1)	69 (89.6)	62 (80.5)	

BMI = Body Mass Index, DLMO = Dim Light Melatonin Onset, DBT-DLMO PAD = Desired Bedtime –Dim Light Melatonin Onset Phase Angle Difference, CGI = Clinical Global Impressions scale, cMEQ = Composite Morningness-Eveningness Questionnaire; Data is represented as mean ± SD.

*Average objective bed and wake times over 5–7 days.

^˄^Reduced n.

### Phase predictions

Previously, others have used a cutoff of ± 1.5 h error between actual and predicted DLMO (3 h range) to determine accuracy of phase predictions^26^. Permitting such a wide range to be considered as a successful prediction (3 h from a total population range of 7.7 h, N = 154) may not be optimal for practical use. We therefore based our assessment of prediction accuracy on a 2 h range, i.e., within ± 1 h of actual DLMO.

Performance of the default dynamic model (i.e., the model previously validated against healthy participants) had root mean square error (RMSE) of 83 min using the default 60-min epochs for light data. Performance of the dynamic model was improved by training on the DSWPD dataset, which resulted in selection of the following optimal parameters: τ = 24.4 h for intrinsic circadian period; k = 0.45 to determine shape of the PRC; G = 37 to determine amplitude of the PRC; maximum allowed missing data interval of 2 h, using mean of previous 2 h for filling missing intervals; and binning light in 60-min windows to the maximum value within the bin.

Performances of the statistical and dynamic models on training and test datasets, respectively, are summarized in Table 2. On the test dataset, the mean absolute errors for the statistical model and dynamic model were 44 and 57 min, respectively. The RMSE for the statistical model and dynamic model were 57 and 68 min, respectively. The statistical model predicted 39% within ± 30 min, 75% within ± 1 h, and 96% within ± 2 h of actual DLMO, while the dynamic model predicted 25% within ± 30 min, 58% within ± 1 h, and 94% within ± 2 h. Actual DLMO was significantly correlated with both the statistical model test predictions (R^2^ = 0.61, *p* < 0.001) and the dynamic model test predictions (R^2^ = 0.48, *p* < 0.001). The addition of advance and delay light regions, based on average DLMO timing, significantly increased the amount of variance explained compared to when only habitual bed or wake time were included in the regression model (R^2^ = 0.49 vs. R^2^ = 0.61).

**Table 2:** Model summary with count and percent of predictions within ± 0.5 h, 1 h, 1.5 h, 2 h and greater than 2 h for the statistical and dynamic model training and tests datasets.

Statistical model								
	Training dataset (n = 77)				Test dataset (n = 77)			
Mean error ± SD (min)	0.02 ± 52.51				− 4.39 ± 56.87			
Mean absolute error ± SD (min)	39.62 ± 34.15				44.58 ± 35.21			
Root mean square error (min)	52.17				56.67			
R	0.766				0.778			
R^2^	0.587				0.605			

	n	n Cumulative	%	%Cumulative	n	n Cumulative	%	%Cumulative
Predictions within ± 0.5 h	37	37	48.05	48.05	30	30	38.96	38.96
Predictions within ± 1 h	25	62	32.47	80.52	28	58	36.36	75.33
Predictions within ± 1.5 h	9	71	11.69	92.21	12	70	15.58	90.91
Predictions within ± 2 h	4	75	5.2	97.4	4	74	5.2	96.1
Predictions > 2 h	2	77	2.6	100	3	77	3.9	100

Dynamic model								
	Training Dataset (n = 76)				Test Dataset (n = 77)			
Mean Error ± SD (min)	− 1.22 ± 63.15				− 4.11 ± 67.91			
Mean Absolute Error ± SD (min)	47.53 ± 40.20				48.40 ± 40.20			
Root Mean Square Error (min)	62.74				67.60			
R	0.655				0.692			
R^2^	0.430				0.479			

	n	n Cumulative	%	%Cumulative	n	n Cumulative	%	%Cumulative
Predictions within ± 0.5 h	29	29	38.16	38.16	19	19	24.68	24.68
Predictions within ± 1 h	24	53	31.58	69.74	26	45	33.76	58.44
Predictions within ± 1.5 h	13	66	17.11	86.84	18	63	23.38	81.82
Predictions within ± 2 h	5	71	6.58	93.42	10	73	12.99	94.81
Predictions > 2 h	5	76	6.58	100	4	77	5.19	100

Examination of the correlations between actual and predicted DLMO between the training and test datasets within each approach showed that the training and test predictions did not differ significantly from one another for either approach (statistical model, Z = 0.18, *p* = 0.86; dynamic model, Z = 0.41, *p* = 0.68). A comparison of the correlations between the two models for test predictions also showed no significant difference (statistical model vs. dynamic model, Z =  − 1.15. *p* = 0.25). Similarly, the slopes of the regression lines for the two models did not significantly differ (slope for statistical model = 0.60 vs. slope for dynamic model = 0.62, *p* = 0.85).

As a comparative method, DLMO was also predicted by subtracting 2 h from actigraphically derived bedtime (averaged over 5–7 days) for each participant, which is reported to be the average phase angle between DLMO and habitual bedtime in a healthy population^18–20,27^. DLMO predicted in this way significantly correlated with actual DLMO (R^2^ = 0.40, *p* < 0.001), with RMSE of 129 min. When this correlation was compared with the final models, there was a significant difference to the statistical model (Z =  − 0.02, *p* < 0.05), but not the dynamic model (Z =  − 0.64, *p* = 0.52).

**Figure 1:**
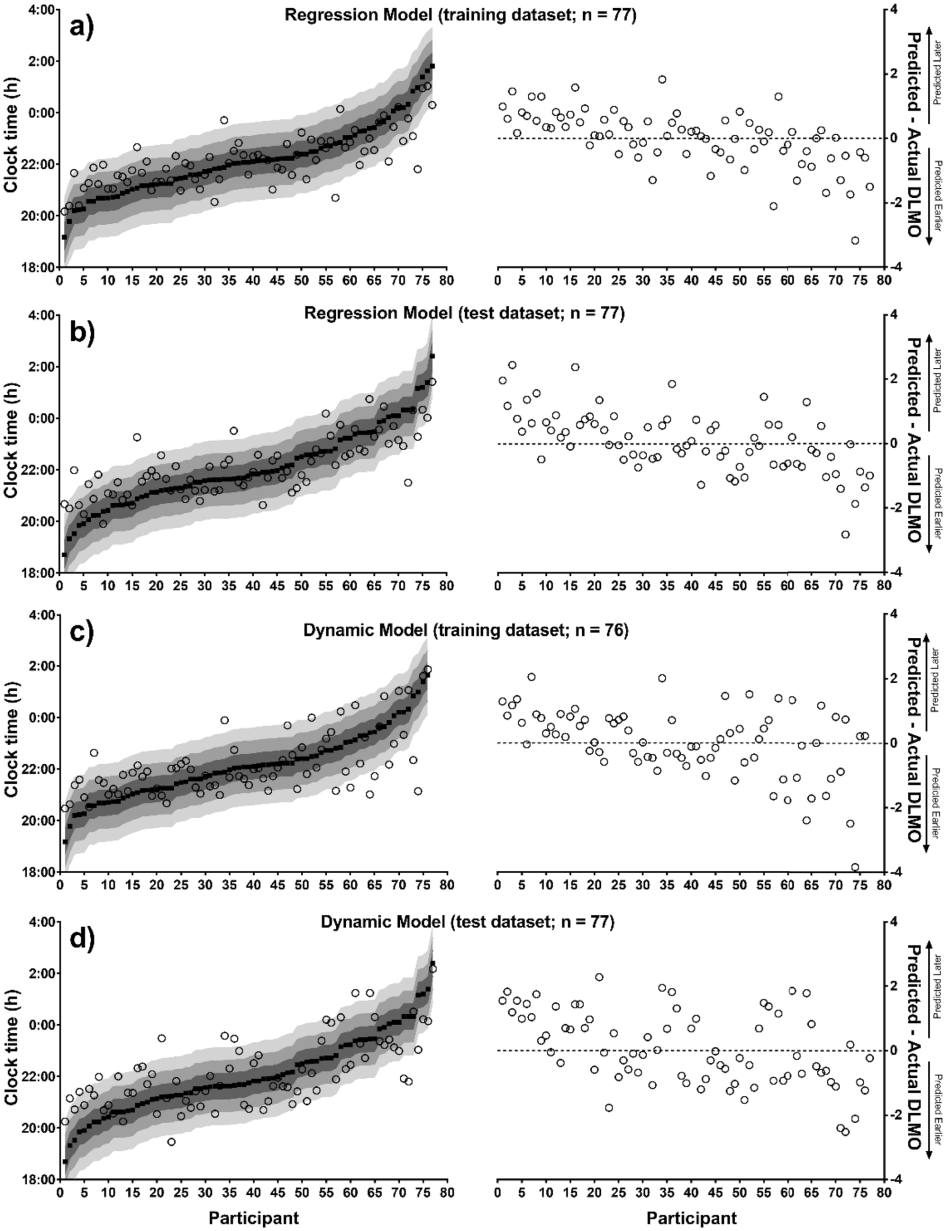
Distribution of actual DLMO time with corresponding predicted DLMO time. Left: Actual DLMO (black squares), ranked from earliest to latest, with corresponding predicted DLMO (open circles) and; Right: error of predictions (0 represents actual DLMO) ranked in the same order, for the statistical model training (panel **a**), and test (panel **b**) datasets, and the dynamic model training (panel **c**) and test (panel **d**) datasets. Prediction error indicated by shaded areas:   =  ± 30 min,   =  ± 1 h,   =  ± 1.5 h.

Figure 1 shows predicted and actual DLMO for each participant for each method, with actual DLMO ranked from earliest to latest onset time. Both methods illustrate a tendency to underestimate population variability, regressing very early or very late individuals towards the population mean.

A comparison was made to determine whether the same participants were being predicted within ± 1 h between the two models (Table 3). Combining the training and test datasets, there were 93 (60%) participants who were predicted within ± 1 h in both models, 32 (21%) participants whose predictions fell in different categories between the dynamic and statistical models, and 28 (18%) participants who were inaccurately predicted (> 1 h error) by both models. There were no significant differences in functional outcomes (mood and daytime function, illness severity) or circadian phase (DLMO time, DBT-DLMO phase angle) between the 28 participants who were inaccurately predicted by both models and the 93 participants who were accurately predicted by both models within ± 1 h.

**Table 3:** Number of participants (N = 153) accurately predicted in both approaches (grey highlight).

		Dynamic model	
		Within ± 0.5 h	> ± 0.5 h
Statistical model	Within ± 0.5 h	32	35
	> ± 0.5 h	16	68
		Within ± 1 h	> ± 1 h
	Within ± 1 h	93	27
	> ± 1 h	5	28
		Within ± 1.5 h	> ± 1.5 h
	Within ± 1.5 h	126	14
	> ± 1.5 h	3	10
		Within ± 2 h	> ± 2 h
	Within ± 2 h	141	7
	> ± 2 h	3	2

As a test of the potential clinical utility of these methods, predicted DLMO was used to classify participants as circadian or non-circadian DSWPD using criteria previously described^14^. Briefly, a circadian classification was given if DLMO occurred 30 min before, or any time after, desired bedtime, while a non-circadian classification was given if DLMO occurred more than 30 min prior to desired bedtime. Performance of the two models in the test dataset was comparable, with similar numbers of true positives, true negatives, false positives, and false negatives (see Table 4). The sensitivity for predicting group classification was 74% for the statistical model and 64% for the dynamic model; specificity was 63% for the statistical model and 66% for the dynamic model. The F1 scores were 0.71 and 0.65 for the statistical and dynamic models, respectively.

**Table 4:** Sensitivity, specificity, F1 scores and accuracy of the final (test) models for group classification (circadian/non-circadian) using predicted DLMOs (true positives = circadian, true negatives = non-circadian).

	Statistical model	Dynamic model
Sensitivity	74.4%	64.1%
Specificity	63.2%	65.8%
F1 Score	0.71	0.65
Accuracy	0.69	0.65

## Discussion

State-of-the-art methods for predicting circadian timing have yet to be tested in clinical populations. We evaluated and compared a novel statistical regression model and a previously validated dynamic model for predicting DLMO time in a clinically diagnosed DSWPD patient population. Both methods for performed similarly. The statistical model accounted for a slightly greater proportion of the variance in actual DLMO time than the dynamic model (60% vs. 48%), but the statistical model also required knowledge of the average DLMO time for the sample to define its PRC regions. The two models were similarly accurate for classifying circadian vs. non-circadian DSWPD (69% vs. 65%). Both models were moderately reliable in predicting circadian phase to within ± 1 h (75% and 58%).

Our study showed that a regression model using mean light exposure during the delay and advance portions of the human PRC, in combination with sex, age, bed/wake times, and chronotype, could predict DLMO significantly more accurately than using bed/wake times alone. Self-reported sleep timing information has previously been used in regression analyses to predict circadian phase in DSWPD, whereby the combination of variables accounted for 77% of the variance in DLMO^26^. The previous study, however, achieved only 79% of predictions within ± 1.5 h, whereas 91% of our regression model estimates were within ± 1.5 h. Additionally, a recent study in which the temporal stability of the melatonin profile over time was examined in 8 DSWPD patients and 5 healthy controls found that DLMO time could be accurately predicted using wake time and the time of morning light onset^28^ Specifically, average actigraphically derived wake time and light onset (averaged across the 3 days prior to DLMO) explained 89% of the variance in DLMO timing. Similar findings were reported by Crowley and colleagues^29^, who estimated DLMO in an adolescent population with healthy sleep. Three separate regression analyses were conducted in which the independent variables were either bed, wake, or mid-sleep times. DLMOs were predicted to within ± 1 h for 78–82% of participants using bedtime, 82–86% using mid-sleep time, and 80–81% using wake time. Our application of a similar statistical model for estimating DLMO in an adult, patient population with a larger sample extends these previous studies, demonstrating the generalizability of such an approach to a clinical setting. Additionally, the current study supports the potential use of objective measures of light exposure, in addition to bed and wake times, for estimating circadian phase timing. The combination of these findings indicates not only that simpler, less invasive methods for estimating circadian phase are a viable alternative, but also that the addition of light is valuable for improving prediction accuracy.

While the statistical model we present here appears promising, it requires further validation before it is to be generalized to clinical settings. The current implementation of the statistical model requires knowledge of the group average DLMO time, which may not be known. Furthermore, the average DLMO time in our sample of DSWPD patients was relatively early compared with other published studies in DSWPD patients. Applying this average DLMO time to other samples could therefore lead to less accurate predictions with the statistical model. In future work, the statistical model should be tested in other DSWPD samples to determine its accuracy, and to determine whether the new sample’s average DLMO time must first be determined. In practice, this DLMO testing could be performed in a subsample to obtain an estimate of the average timing, rather than needing to test the entire sample.

The dynamic model of the circadian pacemaker was originally developed and validated under lab conditions in healthy participants^3,4^. It has since been demonstrated to also perform accurately in healthy participants in the field under a range of conditions^30,31^, making it the best validated model of the human circadian clock^2^. To date, however, it has not been tested in any patient populations. Individuals with DSWPD appear, on average, to have physiological predispositions to phase delay, including a longer intrinsic circadian period^32^ and increased sensitivity to phase-delaying light^33^. Consistent with this, we found that the default model (i.e., using parameters fit against healthy individuals) predicted DLMO time systematically too early. When we allowed parameters to be refit against the Training dataset, we found that a significantly better fit was obtained for DSWPD patients by lengthening the intrinsic circadian period and by slightly increasing the size of the phase-delay region of the phase response curve. With these modifications, the accuracy of the model (RMSE = 68 min) was similar to that reported in the field in healthy populations^2^.

There are many proponents for circadian phase assessments to occur in a clinical setting for diagnosis of circadian rhythm sleep disorders^16,34–36^. It is acknowledged, however, that there are practical barriers to this occurring, including the costs and logistical difficulties involved in conducting DLMO assessments^21^. The results of the current study show that, in addition to predicting actual DLMO timing for the majority of patients within ± 1 h, both models perform equally well at classifying patients with circadian DSWPD^14^. Currently there is no simple method for clinicians to determine if and when a DLMO assessment should be conducted for diagnosis of DSWPD. The models presented here could be valuable clinical screening tools to classify patients as high or low risk for circadian misalignment, enabling efficient identification of those who need further formal phase assessment. Moreover, they could be used to help optimize the timing of interventions, such as light^37^ or melatonin^38^.

For both of the models that we tested, we observed that accuracy tended to be highest for individuals with DLMO times close to the group average, and less accurate for individuals with more extreme DLMO times. This same phenomenon has been reported in other applications of phase prediction models^2^. This tendency for models to regress towards the mean and underestimate population variability is a natural consequence of them being fit at the group, rather than the individual, level. Using an average DLMO time for the entire sample to calculate an average CBTmin time (for the statistical model), as well as the assumption that this population has normal phase relationships between DLMO and sleep or CBTmin (for both models), would both tend to increase accuracy for individuals who are closer to the average. In defining the light bins relative to average DLMO, participants whose actual DLMO occurred closer to the average likely had the most accurately defined lights bins. Similarly, the dynamic model has physiological parameters (e.g., tau) that are fit at the group level. Individuals whose physiology is closer to the average would therefore be likely to have their circadian timing predicted more accurately than for individuals with more extreme physiological parameters. Individuals who are more extreme in their physiological parameters may also experience great intra-individual differences in circadian timing^39^, which could contribute to less accurate predictions. Improved individual-level estimates of model parameters are likely to yield more accurate predictions for individuals who deviate further from the population mean.

There are a number of ways to potentially improve the prediction accuracy of the dynamic and statistical models. These include consideration of inter-individual differences in light sensitivity^40,41^, which have been theoretically demonstrated as a means of capturing inter-individual differences in DLMO time^42^. In defining the light inputs for the statistical regression model, we assumed that the DSWPD population has a similar light phase response curve to the healthy population. There is evidence that patients with DSWPD may have a hypersensitivity to evening light^33,41^. Establishing the light phase response curve in a DSWPD population could therefore help to improve the models. Using an average DLMO time for the entire sample to calculate an average CBTmin time (for the statistical model) and the assumption that this population has normal phase relationships between DLMO and sleep or CBTmin (for both models) are also potential areas of improvement. Although there is some suggestion that phase angle is not altered between sleep and circadian phase markers in DSWPD patients when allowed to sleep at their habitual times compared to healthy individuals^11^, it has not been well characterized, particularly when bed and wake times are shifted to an earlier desired time. Finally, both models were based on photopic illuminance measured at wrist level, which is known not to accurately represent the effect of light on the circadian pacemaker under all conditions^43,44^. Presently, wrist-based actigraphy remains the standard method for collecting patterns of light exposure, but an alternative method that measures light closer to the eye and measure impacts on all photoreceptors in the circadian phototransduction pathway could help improve prediction accuracy^45^.

A measure of circadian timing is important for the diagnosis and appropriate treatment of DSWPD in a clinical setting. Here we have shown that modeling approaches that rely on light–dark and sleep–wake information produce reasonably accurate estimates of circadian phase in up to 75% of participants. Both the dynamic and statistical models show good utility as screening tools in DSWPD and use information that is already routinely collected in diagnostic approaches for DSWPD and other circadian rhythm sleep disorders.

## Methods

This study was conducted as part of the delayed sleep on melatonin (DelSoM) Study (ACTRN12612000425897)^14,46^, a randomized controlled trial testing the efficacy of exogenous melatonin for DSWPD. As the study was a multi-center trial, approval was given by the following human research ethics committees: Monash University Human Research Ethics Committee, The University of Sydney Human Research Ethics Committee, Southern Adelaide Clinical Human Research Ethics Committee, and The University of Adelaide Human Research Ethics Committee. All research was performed in accordance with the ethical regulations and practices stipulated by each ethics committee. Written informed consent was provided by participants prior to study engagement and they were reimbursed for study-related expenses^14,46^.

### Participants

Data from this study have previously been reported^14,46,47^. Briefly, data were included from 182 (89 M, 93 F) participants with clinically diagnosed DSWPD. Participants were recruited at three study sites (Melbourne, Sydney, Adelaide) from the community via radio, newspaper, and poster advertising, and from clinics through referrals from sleep physicians and psychologists.

Participants were aged 16–65 years, with a body mass index > 18 and < 35 kg/m^2^. Additionally, participants were required to work or study ≥ 5 consecutive days each week. Participants who reported any of the following were excluded: comorbid sleep disorder (except insomnia); drugs of abuse or concurrent medication likely to affect sleep; history of psychiatric disorder in the past 12 months, other than depression; caffeine consumption > 300 mg per day; alcohol consumption > 14 standard drinks per week; history of substance abuse in past 12 months; investigational drug use in past 60 days; pregnancy or lactation; night shift work in past 6 months; transmeridian travel in the past 2 months; allergies to any medicines, foods, preservatives, or dyes; and liver, kidney, or autoimmune disease. More detailed inclusion and exclusion criteria have been reported elsewhere^14^.

### Screening

Potential participants completed a preliminary online eligibility questionnaire to assess risk of DSWPD. Those deemed as high risk completed further screening after providing consent. Participants were assessed by a sleep physician to confirm a diagnosis of DSWPD, according to current International Classification of Sleep Disorders diagnostic criteria at the time of study^48^, and also completed questionnaires relating to health and lifestyle, sleep habits, mood, and daytime function. Participants also completed the Composite Morningness-Eveningness Questionnaire (cMEQ)^49,50^, a 13-item questionnaire measuring an individual’s preferences for the timing of mental and physical activity.

### Sleep/wake and light assessment

Participants recorded sleep and wake times at home for 7 days using a sleep diary and wrist actigraphy (Actiwatch-L, Actiwatch-2, or Actiwatch Spectrum; Philips Respironics, Bend, OR, USA). Participants were instructed to maintain their normal sleep–wake schedules and record bed and wake times, time to fall asleep, and any awakenings after sleep onset each morning upon waking in the sleep diary provided.

Actigraphy devices, worn on the non-dominant wrist, were used to obtain light (white light, lux) and activity measures in 1-min epochs. Software sensitivity was set to medium (40 activity counts/minute) to determine each 1-min epoch as sleep or wake (Actiware 5 software, Philips Respironics Inc, Bend, OR, USA). To limit loss of light data, participants were instructed to keep the device uncovered (e.g., by sleeves) at all times. Bed and wake times in actigraphy were identified using the times reported in sleep diaries. In the case of discrepancies between sleep diaries and actigraphy, the following process was applied: if subjective bedtime was reported as ≥ 60 min before a substantial reduction in activity and light levels, bedtime was adjusted to the time of decrease in activity and light. If reported wake time was ≥ 60 min after a substantial increase in activity and light, wake time was shifted to the start of the sustained activity and light increase. These timings were determined via visual inspection by an independent researcher and then reviewed by two study researchers for consensus (JM and MM). From these data, bedtime and wake time were computed for main sleep episodes^14,46^.

For both the dynamic and statistical models, white light (photopic illuminance) was used^51^. Light data were extracted from the adjusted actigraphy data files in 1-min epochs for each participant. Light data were further cleaned, with removal of data with values < 1 lux during wake, as it was assumed that this was likely due to the device being covered^52^. Additionally, light levels during sleep were set to zero, as the eyelids are closed during sleep, minimizing retinal light exposure^53^.

### Circadian phase assessment

Immediately following the 7 days of at-home sleep/wake and light assessment, participants attended a laboratory session to undertake a circadian phase assessment measuring salivary dim light melatonin onset (DLMO). Participants arrived at the laboratory 6 h prior to their subjectively reported habitual bedtime and following admission, were seated in dim lighting (< 3 lux). From 5 h prior to habitual bedtime until 2 h after habitual bedtime, saliva samples were taken every hour. DLMO was calculated as the time that melatonin concentrations crossed and remained above a threshold of 2.3 pg/mL, calculated from linear interpolation between the samples immediately before and after the threshold^54^. Details of this procedure have been reported previously^14,46^.

### Dynamic model

The dynamic model used a mathematical model that has been previously tested and validated against data from carefully screened healthy humans in laboratory conditions^4^, as well as in field conditions^10,30^. The model includes light processing in the retina by photoreceptors, the effects of light (photic drive) and the effects of sleep/wake patterns (non-photic drive) on the circadian clock, and a limit-cycle oscillator model of the central circadian clock. For the full model equations and parameter values, see the original publication^4^.

The model includes the following equation for the photic drive to the circadian pacemaker

$$B = G\alpha \left( {1 - n} \right)\left( {1 - bx} \right)\left( {1 - by} \right),$$

where $G$ is a constant of proportionality determining amplitude of the light PRC, $\alpha \left( {1 - n} \right)$ is the rate at which retinal photoreceptors are being activated, $b$ is a constant determining the overall shape of the light PRC, and $x$ and $y$ are the variables of the circadian pacemaker. All parameters of the model have been previously fit against healthy participant data. The model generates predicted times of minima in the endogenous core body temperature rhythm. DLMO is estimated to occur 7 h before these minima, which is based on the average phase angle difference between DLMO and CBTmin^55^.

Here, the model was first used in its default form to predict DLMO times for all participants to determine whether the model fit against healthy participants could generalize to this patient population. In other papers that have used versions of the Jewett-Kronauer model, light data have either been input in 1-min epochs^10^ or set to the maximum value in 60-min epochs^40^. A range of epoch choices were therefore used here, with 60-min epochs for testing default parameters.

Subsequently, the model was trained against the DSWPD dataset to determine if model performance could be improved. The dataset was split into training (50%) and test (50%) sets, split evenly within each of the three test sites. For training, the following modifications were permitted: (1) changes in the intrinsic circadian period in steps of 0.05 h, allowing values equal to or above the default 24.15 h, given empirical evidence for longer tau in this population^32^; (2) changes in the parameter $b$, in steps of 0.05, allowing values equal to or above the default 0.40, given empirical evidence for greater phase delays in this population^33^; (3) changes in the parameter $G$,in steps of 5%, allowing values equal to or greater than the default 37, given empirical evidence for greater light sensitivity in this population^33,41^; (4) binning of light data in 1, 2, 5, 10, 15, 30, 60, or 120 min bins, to either the mean value or maximum value in the bin; (5) since the model requires continuous light data with no missing values, we allowed missing intervals to be filled with either a value of 0 lux or with the average value for the previous 2 h. The maximum allowed gap length was allowed to be in the range 1–6 h, in steps of 1 h. The longest continuous portion of the light time series with no missing values was then used as input to the model. No other changes to model parameters or equations were made. The non-photic component of the model was driven by sleep/wake state. For each participant, light data were input repeatedly for 60 days, allowing the model to approach a steady cycle, as in previous papers using the model^10,31^. Predicted DLMO time was the average predicted DLMO time across the final input cycle.

### Statistical model

**Figure 2:**
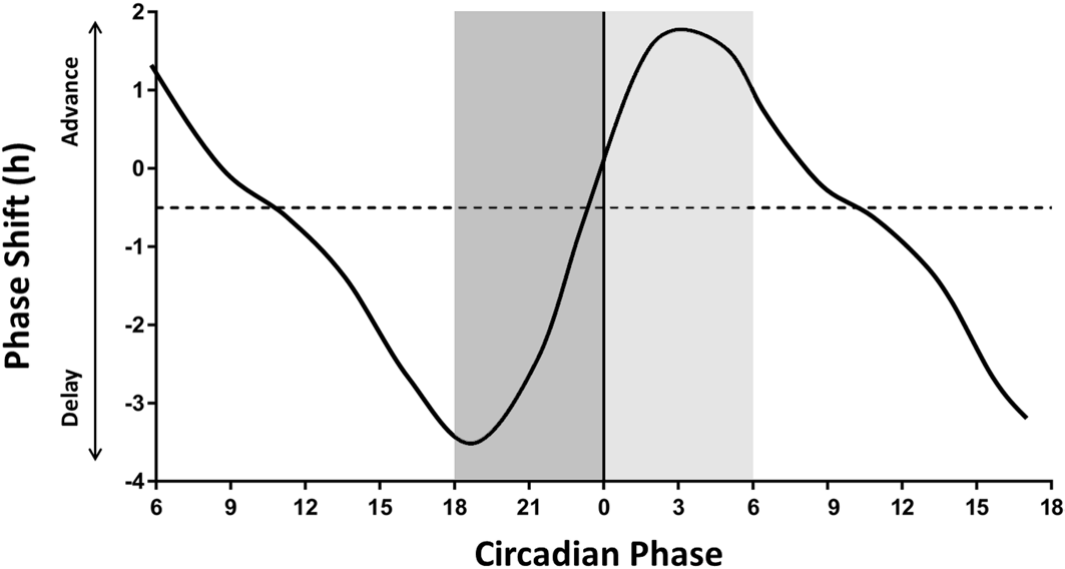
Phase response curve for light. A reference phase response curve (based on^50^) was used for determining phase in the regression model. Phase shifts are plotted (solid black line) against the circadian timing of light exposure. Positive values on the y-axis indicate a phase advance and negative values indicate a phase delay. Zero on the x-axis represents core body temperature minimum. For the current study, the dark grey bar indicates the “delay zone”, which encompasses the circadian time at which light will induce a maximal phase delay. The light grey bar represents the “advance zone”, and encompasses the time at which light will induce a maximal phase advance.

A statistical model for predicting circadian phase was developed based on a multiple linear regression analysis, with DLMO as the dependent variable. The following predictors were included: (1) light data from the advance and delay portions of the PRC (Figure 2); (2) demographic information (age, sex); (3) actigraphically derived bedtime and wake time and; (4) composite morningness-eveningness score (cMEQ)^50^. Variables (2)–(4) were included in the model as covariates based on previous reports that these variables are associated with circadian phase^56–59^. All variables, with the exception of sex and cMEQ, were continuous variables. cMEQ was treated as a categorical variable with two levels: extreme evening type (scores between 16–30) and moderately evening type (scores between 31–41). The statistical model was applied to the same training and test datasets as those described for the dynamic model above.

To quantify the time points at which light would have maximal circadian effects, we used a PRC for light^55^ (Fig. 1) and classified the 6 h prior to core body temperature minimum (CBTmin) as the interval corresponding to the delay zone and the 6 h after CBTmin as the advance zone. Light data were log_10_-transformed, as has been done by others previously, to account for brief periods of high light exposure that may have skewed the data^52^ and zero values were assigned a value of 0.001 lux to allow transformation. As we did not have an actual measure of CBTmin in this study, to determine CBTmin we assumed a phase angle between DLMO and CBTmin of 7 h, as reported in other studies^55,60,61^. CBTmin was thus estimated by adding 7 h to the average DLMO time for the total sample (N = 154); average DLMO time for the sample was 22:10 h, and average estimated CBTmin time was 5:10 h. This CBTmin time was then applied to all participants to determine the delay and advance zones. Average light exposure in the delay and advance zones was then calculated for each individual on each day with valid data (minimum 5 consecutive days, maximum 7 consecutive days; 6.83 ± 0.47 days). The average delay and advance light zones from each day were then averaged across the days of recording, with each participant having one measure of delay light and one measure of advance light.

### Data analysis and statistical methods

Matlab R2018a (Natick, Massachusetts) was used for the dynamic model simulations, using ode15s for solving differential equations and using a custom grid search method for finding optimal parameter values. SPSS Statistics Version 20.0 (IBM, Armonk, New York) was used for the statistical model and all other data analysis. Data are expressed as mean ± standard deviation (SD) unless otherwise stated. Significance level was set at 0.05. Variables were compared between training and test datasets using a chi-squared (goodness of fit) test or independent samples t-test. To evaluate the predictive performance of each method, the mean error, mean absolute error, and root mean square error of the difference between actual DLMO and predicted DLMO were calculated. Pearson’s correlation coefficients were computed between actual DLMO and predicted DLMO for the dynamic and statistical models and regression lines were fit for each correlation. To determine whether there were significant differences between Pearson’s correlation coefficients for the dynamic and statistical models, a Fisher’s r to z transformation was used. The regression slopes were also compared to determine whether there were significant differences between models. We additionally calculated the percentage of participants for whom predicted DLMO was within ± 30, ± 60, ± 90, ± 120, or > 120 min of actual DLMO.

The predictive value of each model was also assessed by classifying participants as “circadian” or “non-circadian” DWSPD, based on a previously published classification scheme^14^. Circadian DSPWD participants were defined as individuals whose DLMO time was within half an hour before, or any time after, desired bedtime, while non-circadian DSPWD participants were defined as those whose DLMO occurred more than half an hour before desired bedtime^14^. Sensitivity, specificity, F1, and accuracy scores were calculated for both models.

### Data retention

Out of 182 participants, 28 participants (15%) were excluded. Participants were excluded from the dynamic modeling approach, due to (1) irregular actigraphy data (n = 3) in which the pattern of light exposure and/or activity was highly irregular with no discernable diurnal rhythm, or (2) actigraphy recordings that did not occur in the 7 days immediately prior to the circadian phase assessment (n = 25).

To ensure comparability between the dynamic and statistical models, the same participants were excluded from the statistical model. As such, the total number of participants included was 154. One participant was excluded from the dynamic model test dataset due to inability to construct at least 1 day of consecutive valid light data due to gaps in the dataset.

## Data Availability

Materials and data in this publication can be requested via the CRC for Alertness, Safety and Productivity (Alertness CRC) by emailing inquiries@alertnesscrc.com.
